# Retraction: Do Magnetic murmurs guide birds? A directional statistical investigation for influence of Earth’s Magnetic field on bird navigation

**DOI:** 10.1371/journal.pone.0351890

**Published:** 2026-06-22

**Authors:** The PLOS One Editors

After publication of this article [1], concerns were raised about the validity of the reported work and about the presence of incomplete sentences and other anomalies within the body text. The *PLOS One* Editors consider the text issues arose from apparent errors in the underlying LaTeX code and this aspect of the concerns was considered resolved.

In editorial follow-up, a Statistical Advisor noted concerns about the validity of the assumptions, model and reporting in the article [1], which the *PLOS One* Editors discussed with the corresponding author. Specifically,

- The study’s assumption that consecutive changes in direction (turning angles) are independent appears unjustified. The corresponding author stated that turning angles were treated under an independence assumption as an approximation, but noted consecutive turning angles from tracked trajectories may exhibit serial dependence because adjacent fixes belong to the same movement path. The Statistical Advisor advised that violation of the independence assumption may severely bias the subsequent statistical analyses.
• Eqs 20 and 21 in [1] appear to define linear models on circular data, which do not respect the topology required for a valid circular-circular regression model. The corresponding author clarified that Eqs 20 and 21 serve as shorthand for a cosine-sine conditional mean framework introduced earlier (Eqs 13–19), where circular topology is respected by reconstructing fitted angles via atan2(g2, g1), and that the equations should not be interpreted as stand-alone circular-circular regressions.

The Statistical Advisor advised that Eqs 13–19 are acceptable for the specification of a circular regression model; however, the regression analysis was not accompanied by an appropriate analysis of residuals.

- The Algorithm 1: Bird Migration Analysis Workflow subsection of the Materials and Methods section describes a workflow that involves the von Mises mixture model and a spherical mixture model. None of these models appear to have been estimated and used in the data analysis.
- Reporting of results is incomplete, limiting their interpretation and reproducibility:
  - Table 1 omits significance levels, von Mises parameter estimates, and p‑values.
  - Tables 2-3 report only a single summary statistic (*ρ*), omitting the fitted coefficient vectors, standard errors, and model basis, the inferential framework needed for a regression table.
  - Tables 4-5 omit p‑values, sample sizes, and clear test descriptions.

The corresponding author noted that the article was intended as an introductory study presenting a novel idea and its initial validation, with more comprehensive analyses left for future work.

The *PLOS One* Editors consider that the concerns about model assumptions, specification, and reporting are not resolved. In light of these concerns, which call into question the validity and reliability of the published results, the *PLOS One* Editors retract this article.

PG agreed with the retraction. DC, AB, and SSD did not agree with the retraction.
